# Is psychological distress associated with carpal tunnel syndrome symptoms and nerve conduction study findings? A case–control study from Syria

**DOI:** 10.1002/brb3.2493

**Published:** 2022-01-18

**Authors:** Aya Alsharif, Aya Al Habbal, Yaman Daaboul, Lama Al Hawat, Osama Al Habbal, Ameer Kakaje

**Affiliations:** ^1^ Faculty of Medicine Damascus University Damascus Syria; ^2^ Diagnostic Medical Center – Yaman Daaboul Neurophysiology Clinic Damascus Syria; ^3^ University Hospital Geelong, Barwon Health Victoria Australia

**Keywords:** anger, carpal tunnel syndrome, functional disability, nerve conduction studies, psychological status, symptom severity

## Abstract

**Background:**

Carpal tunnel syndrome (CTS) is a common entrapment neuropathy of the median nerve at the wrist which causes severe symptoms. However, psychological aspects can affect patients' perception of this pain and can cause similar pain in some instances. This study aims to determine the association between symptoms severity, functional status, and nerve conduction studies (NCS) of adult patients with CTS and their anger, anxiety, and depression status.

**Methods:**

This case–control study was conducted in clinics in Damascus, Syria. Controls were frequency matched by gender and age from a general clinic. Interviews based on questionnaires were used that included the Boston Carpal Tunnel Questionnaire (BCTQ‐A), Hospital Anxiety and Depression Scale (HADS), Dimensions of Anger Reactions Scale‐5 (DAR‐5), and NCS.

**Results:**

Overall, 242 patients (121 cases) were included in this study. Cases with CTS had significantly higher anxiety and depression when compared to controls, but not higher anger. Cases with higher anxiety, depression, and anger had significantly more CTS symptoms and less functional status. Anxiety was also higher in cases with normal NCS in the case group. When using regression, anxiety and depression remained significantly associated with having CTS.

**Conclusion:**

Anxiety and depression are more prominent with CTS. Furthermore, having anxiety and depression were associated with more CTS symptoms in the hand. Having anger was also associated with more CTS symptoms among cases. These findings emphasize the importance of psychological aspects when having hand pain or CTS symptoms as these patients might have these symptoms despite having normal NCS.

## Graphical abstract

Cases with carpel tunnel syndrome (CTS) have more psychological burden than control.

CTS symptoms and disability are associated with more severe anxiety, depression, and anger.

Having anxiety and/or depression with CTS are associated with more normal nerve conduction studies which suggests that having psychological burden might provoke symptoms that mimic CTS.

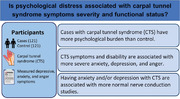

## 1 INTRODUCTION

Carpal tunnel syndrome (CTS) is a common entrapment neuropathy of the median nerve at the wrist (Demino & Fowler, 2021). Much as the name implies, the median nerve is compressed when entering the carpal tunnel as it travels beneath the transverse carpal ligament (Bhatt et al., 2015). Though the underlying cause of the compression can be idiopathic, it can be attributable to a number of different factors such as malpositioning of the wrist, any condition that leads to an excess fluid within the body that leads to an increased volume in the tunnel, and masses that could occupy the volume of the tunnel (Genova et al., 2020).

The nerve damage from the compression produces sensory symptoms, such as numbness, tingling, and pain within the hand. As the disease progresses, soft tissue injury occurs, and disabling motor symptoms such as reduction of grip strength become apparent. This impairment may progress into thenar atrophy in advanced cases (Bhatt et al., 2015; Genova et al., 2020; McCallum et al., 2019).

These symptoms mostly aggravate at night and have a huge negative impact on the ability to work and perform daily activities leading to increased morbidity and inability to attend work (Demino & Fowler, 2021; Genova et al., 2020; McCallum et al., 2019). They can also affect patients’ quality of life and cause a deteriorating in mental health (Jerosch‐Herold et al., 2017; McCallum et al., 2019). Moreover, psychological distress itself can intensify symptom perception, or even produce subjective symptoms resembling CTS (McCallum et al., 2019; Papadopoulou et al., 2021). The prevalence of anxiety and depression in CTS patients is 28.7% and 37.6%, respectively, which is higher than the general population (Filho et al., 2020; McCallum et al., 2019). In addition, the prevalence of CTS is higher among women, usually after their sixth decade of age (Basiri & Katirji, 2015). It is speculated that decreased estrogen antinociceptive effect caused by the decreased estrogen concentrations is the reason behind the increased likeliness of anxiety and depression in women (Filho et al., 2020).

Since psychological distress affects CTS symptoms, diagnosis work‐up and treatment program, it is important to assess the presence of an association between them as it could affect patients’ outcomes. The more symptomatic the patients are, the higher they scored in anxiety and depression scales even before having a confirmed CTS by electric studies (McCallum et al., 2019). There is still considerable controversy surrounding the association between psychological status and nerve conduction studies (NCS) (Papadopoulou et al., 2021). To our knowledge, there is a lack of studies discussing anger's effect on CTS symptoms and NCS. However, some studies included the association with anxiety and other psychological aspects. The aim of this study is to broaden current knowledge of the association between psychological status such as anger and anxiety and the severity of CTS symptoms along with NCS. This is the first study to include anger in CTS psychological factors' assessment.

It is noteworthy to mention that we undertook this study in Syria that has been in war for ten years. Therefore, by taking into account the effects of war environment on mental health, our study provides special insights on CTS in war time. As high prevalence of mental health disorders was found in Syria, particularly during war and COVID‐19 (Levine et al., 1993), we speculated that they both might have indirectly led to an increase of CTS symptoms as well. This is a case–control study that aims to determine the association between symptoms severity, functional status, and NCS of adult patients with CTS and their anger, anxiety, and depression status. This is the first study to explore into CTS in Syria and its association with mental health during the war and COVID‐19.

## 2 METHODS

### 2.1 Study design

This case–control study was conducted at a neurophysiology outpatient clinic and general outpatient clinics at Al‐Assad teaching hospital in Damascus, Syria. This study was conducted from December 2020 to June 2021. Interviews based on questionnaires were used to collect data after applying the inclusion and exclusion criteria for both, the cases or the controls. The interviewers were trained to facilitate data collection and explain any vague information in the questionnaires to overcome any educational or cultural barrier that might have prevented proper understanding.

### 2.2 Study population

Cases were at least 18 years old, able to give informed consent, and were diagnosed with CTS by a combination of clinical features, physical examination, and NCS findings. We excluded cases that had either a neuropathy other than CTS, such as ulnar neuropathy, or previous CTS surgery during the last year. Cases were frequency matched with a control group by gender and age (not more than ten years range). Controls were selected from the general outpatient clinics at Al‐Assad University Hospital provided that they had no history of previous or current nerve entrapments and did not need neurology symptoms or referrals. We excluded any case or control who had conditions causing chronic pain other than the pain from CTS in the case group.

### 2.3 Questionnaires

Questionnaires included demographics such as gender, age, educational level, work, marital status, number of children, along with handedness and shisha or cigarette smoking.

The evaluation of patients with suggestive CTS symptoms was made through taking a detailed history of their condition, performing a clinical examination, which includes Phalen and Tinel tests, and finally confirming the diagnosis by conducting NCS.

CTS symptoms severity was assessed by asking cases to complete the Arabic version of Boston Carpal Tunnel Questionnaire (BCTQ‐A). Boston Carpal Tunnel Questionnaire (BCTQ) is a self‐report questionnaire that records symptom severity and functional status in CTS patients (Levine et al., 1993). It represents CTS severity in the last two  weeks and consists of two scales; the first one is the symptom severity scale (SSS) that has 11 items that cover six domains include: pain, numbness, nocturnal symptoms, paraesthesia, weakness, and functional status. Its score ranges from 1 to 5, which is the most severe. The second one is the functional status scale (FSS) that has 8 functional activities that are commonly affected by CTS (difficulty in writing, buttoning clothes, opening jars, holding a book, gripping a telephone handle, performing household chores, carrying grocery bags, bathing, and dressing). The scores also range from 1 to 5 that represent not being able to perform the activity at all (Alanazy et al., 2019). Since many patients were illiterate, we had to change the question asking about difficulties of writing or holding a book while reading to difficulties of using cutlery during their meals or holding their plate while eating, respectively.

The Hospital Anxiety and Depression Scale (HADS) was also used which is a brief, clinically meaningful scale that screens for the levels of anxiety and depression in clinics. The limitation of this version is that it does not have somatic symptoms of depression to reduce the effect of the current medical condition(s) on the scores. It consists of two subscales and scores can predict the psychosocial and physical outcomes. HADS has 14 items as anxiety and depression have seven items each. Each item is scored on a four‐point scale ranging from 0 to 3 and the maximum score for each subscale is 21. Scores that range from 0 to 7 are considered normal while 8–10 are considered borderline, and 11–21 are considered as anxiety or depression cases (Terkawi et al., 2017; Zigmond & Snaith, 1983).

Dimensions of Anger Reactions Scale‐5 (DAR‐5) is used to assess problematic anger. It is a mini screening measure of anger reactions for the past four weeks. It assesses the frequency, intensity, and duration of anger, antagonism toward others, and social relations interference. Scores of each item range from 1 (None or almost none of the time) to 5 (All or almost all of the time). Total scores above 12 are considered problematic anger (Forbes et al., 2014; Kakaje et al., 2021).

NCS: Median distal motor latency (DML) and median sensory NCS (between the wrist and digit, between the wrist and palm) are of the best NCS tests to evaluate CTS according to a literature review of the American Association of Neuromuscular and Electrodiagnostic Medicine (AANEM) (Basiri & Katirji, 2015). Of these tests, DML and distal sensory latency (DSL) are the most popular ones, so that we used them along with other tests such as sensory nerve conduction velocity (NCV) and amplitude in order to increase the sensitivity and specificity of our tests. The sensory conduction study was performed by placing an active electrode on the index at a distance of 13–14 cm from a stimulator in the middle of the wrist. We also stimulated the median nerve at the palm. The motor conduction study was done by placing an active electrode on the belly of the abductor pollicis brevis (APB) muscle. We stimulated the median nerve at the wrist at a distance of 4‐6 cm from the active electrode.

Neurophysiological grading of CTS was done based on the study of Bland (2000) and Jerosch‐Herold et al. (2017):

Grade 0: normal (absence of abnormal neurophysiological findings)
Grade 1: very mild (NCV from index to wrist < 50m/s and ≥ 40m/s, DML from wrist to abductor pollicis brevis [APB] > 4.0ms)
Grade 2: mild (NCV from index to wrist < 40 m/s, DML [APB] < 4.5ms)
Grade 3: moderate (DML [APB] > 4.5 ms and < 6.5ms with normal index finger sensory nerve action potential [SNAP])
Grade 4: severe (DML [APB] > 4.5ms and < 6.5ms with absent index [SNAP])
Grade 5: very severe (DML [APB] > 6.5ms)
Grade 6: extremely severe (motor nerve action potential (MNAP) [APB] < 0.2 mV)

### 2.4 Definitions

The classification of clinical symptoms such as numbness, tingling, or pain depended on the location where the patient experiences these symptoms. It comprised 4 categories: classic, probable, possible, and unlikely, which represent the following patterns of symptoms: two of three fingers (thumb, index, and long) without the involvement of the palm or hand dorsum, two of three fingers (thumb, index, and long) and the palm but no confinement to the ulnar side, one of three fingers (thumb, index, and long), and none of three fingers, respectively (Basiri & Katirji, 2015; Radwan et al., 2018).

Tinel's test result is considered positive if patients display CTS symptoms while the examiner is percussing the median nerve over along the carpal tunnel (Basiri & Katirji, 2015; Genova et al., 2020). In comparison, the result of Phalen's maneuver, by which patients flex the wrist vertically, is described as positive if the patient experiences symptoms in the distribution of the median nerve (Basiri & Katirji, 2015; Genova et al., 2020).

Education was categorized according to their years to: illiterate, which means he did not finish year 6 at school or has not entered school altogether, elementary if he finished year 6, primary if he finished year 9, and secondary if he finished year 12.

### 2.5 Statistical analysis

Statistical analysis was performed using the Statistical Package for Social Science (SPSS 25). Descriptive statistics were stated as the mean and standard deviation, frequency and percentage. Chi‐square test (or Fisher's exact test) were performed for comparing categorical data and Student's *t‐*test or one‐way analysis of variance (ANOVA) were used for continuous variables as appropriate. Pearson's correlations were used to evaluate relationships between variables. A *p‐*value of less than .05 was considered significant. Linear forward regression was also used for multivariable analysis of numeric variables (the scores) on significant factors.

### 2.6 Ethics

The study was approved by the local ethics committee of Damascus University, faculty of medicine.

## 3 RESULTS

A total of 242 patients participated in this study, 121 cases and 121 controls. Each group has 96 females and 25 males. The mean age was 43.95 years ± 13.38 in cases and 43.12 years ± 13.19 in controls. There is no difference in the mean age between cases and controls since the groups were frequency matched by gender and age. Most patients in both groups were right‐handed (93.4% in cases, 86% in controls). The predominant level of education was elementary (26.4%) in cases and College education (32.2%) in controls. Housewives were 61.2% in CTS patients compared to 41.3% in controls. Demographic variables were summarized in (Table 1).

**TABLE 1:** Comparison of demographic characteristics between cases and controls

Characteristic	Cases (121)	Controls (121)	*p*‐Value
Age, years			
Mean ±SD	43.95 ± 13.38	43.12 ± 13.19	.629*
Gender			
Women (%)	96 (79.3)	96 (79.3)	
Men (%)	25 (20.7)	25 (20.7)	
Dominant handed			
Right (%)	113 (93.4)	104 (86)	.057**
Left (%)	8 (6.6)	17 (14)	
Education			
Illiterate (%)	25 (20.7)	5 (4.1)	<.001**
Elementary (%)	32 (26.4)	25 (20.7)	
Primary (%)	24 (19.8)	31 (25.6)	
Secondary (%)	20 (16.5)	17 (14)]	
College (%)	20 (16.5)	39 (32.2)	
Masters and PhD (%)	0 (0)	4 (3.3)	
Work			
Jobless (%)	11 (9.1)	14 (11.6)	.004**
Housewife (%)	74 (61.2)	50 (41.3)	
Farmer (%)	5 (4.1)	1 (0.8)	
Teacher (%)	4 (3.3)	10 (8.3)	
Employee (%)	14 (11.6)	20 (16.5)	
Domestic manager (%)	1 (0.8)	0 (0)	
Barber and Tailor (%)	1 (0.8)	9 (7.4)	
Carpenter and painter (%)	4 (3.3)	3 (2.5)	
Health care (%)	1 (0.8)	7 (5.8)	
Merchant (%)	4 (3.3)	6 (5)]	
Driver (%)	2 (1.7)	1 (0.8)	
Marital Status			
Single (%)	14 (11.6)	25 (20.7)	.211**
Married (%)	104 (86)	92 (76)	
Divorced (%)	2 (1.7)	2 (1.7)	
Widow (%)	1 (0.8)	2 (1.7)	
Number of children			
Mean± SD	4.32 ± 3.1	2.88 ± 2.68	<.001*
Cigarette smoking			
Yes (%)	28 (23.1)	27 (22.3)	.878**
No (%)	93 (76.9)	94 (77.7)	
Hookah smoking			
Yes (%)	14 (11.6)	18 (14.9)	.448**
No (%)	107 (88.4)	103 (85.1)	

* Student's *t‐*test.

** Chi‐square test (or Fisher's exact test).

The percentage of symptoms' patterns, as described by CTS patients, are: classic (18.2%), probable (65.3%), possible (6.6%), and unlikely (9.9%). Based on the results from the BCTQ‐A SSS, patients were classified into asymptomatic (1.7%), mild (41.3%), moderate (34.7%), severe (20.7%), and very severe (1.7%). The data were also classified according to BCTQ‐A functional status scale into asymptomatic (13.2%), mild (33.9%), moderate (33.1%), severe (15.7%), and very severe (4.1%). Finally, 57 (47.1%) of CTS patients had a positive Tinel sign, and 45 (37.2%) had a positive Phalen sign.

In the case group, no statistically significant difference was found between left and right hand in terms of NCS except in sensory median nerve amplitude. It is noteworthy to mention that 31.4% of NCS in both hands showed normal results among cases. (Table 2)

**TABLE 2:** Comparison of nerve conduction studies between right and left hands

	Left	Right	*p*‐Value
Distal sensory latency			
Mean ± SD	3.2 ± 0.58	3.3 ± 0.70	.400*
Sensory nerve amplitude			
Mean ±SD	40.35 ± 21.52	34.84 ±2 0.70	.049*
Sensory nerve conduction velocity			
Mean ± SD	44 ± 6.56	43 ± 7.13	.267*
Distal motor latency			
Mean ± SD	3.92 ±.95	4 ± 0.94	.580*
Motor nerve amplitude			
Mean ± SD	11.22 ± 4	11.29 ± 6	.924*
Nerve conduction study grading			
Normal (%)	19 (15.7)	19 (15.7)	.640**
Very mild (%)	70 (57.9)	59 (48.8)	
Mild (%)	10 (8.3)	14 (11.6)	
Moderate (%)	15 (12.4)	18 (14.9)	
Severe (%)	0 (0)	0 (0)	
Very severe (%)	2 (1.7)	4 (3.3)	
Extremely severe (%)	0	0	

* Student's *t‐*test.

** Chi‐square test (or Fisher's exact test).

A statistically significant difference was found between cases and controls in terms of anxiety and depression. In contrast, the mean of the anger scale was not statistically different between the two groups. Both groups have DAR‐5 scores greater than 12. In comparison with cases, the percentages of normal anxiety and depression scores, as well as mild ones, were higher in controls (74.4% for anxiety and 83.4% for depression). Based on the HADS score for anxiety and depression, severe scores were higher in cases in comparison with controls. Psychological factors and their association with CTS are summarized in (Table 3).

**TABLE 3:** Comparison of psychological factors between cases and controls

	Cases	Controls	*p*‐value
HADS‐anxiety			
Mean ± SD	10.12 ± 5.22	7.55 ± 4.77	<.001*
HADS‐depression			
Mean ± SD	9.59 ± 5.25	6.95 ± 3.78	<.001*
DAR‐5 anger			
Mean ± SD	13.97 ± 5.56	12.76 ± 4.53	.065*
HADS‐anxiety grading			
Normal (%)	46 (38)	68 (56.2)	.010**
Mild (%)	20 (16.5)	22 (18.2)	
Moderate (%)	27 (22.3)	17 (14)	
Severe (%)	28 (23.1)	14 (11.6)	
HADS‐depression grading			
Normal (%)	43 (35.5)	69 (57)	<.001**
Mild (%)	27 (22.3)	32 (26.4)	
Moderate (%)	25 (20.7)	16 (13.2)	
Severe (%)	26 (21.5)	4 (3.3)	

*Abbreviations*: DAR‐5, Dimensions of Anger Reactions Scale‐5; HADS, Hospital Anxiety and Depression Scale.

* Independent *t‐*test was used.

** Chi‐square test was used.

The patients with normal NCS had the highest mean of anxiety among other NCS groups. When doing the one‐way ANOVA test, the difference between groups was only statistically significant in the left hand. However, that difference was only significant between the normal and moderate groups after running the post hoc test (Table 4).

**TABLE 4:** Factors associated with the result of nerve conduction study

	Anxiety	Depression
Mean left hand EDX grading ± SD		
Normal	13.47 ± 4.50	11.89 ± 5.12
Very mild	9.97 ± 5.49	9.80 ± 5
Mild	9.60 ± 4.30	8.20 ± 5.15
Moderate	8.53 ± 4.47	8.40 ± 6.03
Very severe	7 ± 1.41	6 ± 7.07
*p‐value*	**.039** ^#^	.193^#^
Mean right hand EDX grading ± SD		
Normal	12.16 ± 4.40	10.42 ± 5.54
Very mild	10.10 ± 5.53	9.61 ± 4.94
Mild	9.86 ± 5.25	11.07 ± 5.39
Moderate	8.61 ± 4.03	6.56 ± 3.91
Very severe	7 ± 0.82	11.25 ± 7.41
*p‐value*	.186**^#^**	.078**^#^**

*Abbreviations*: EDX: electrodiagnostic; HADS, Hospital Anxiety and Depression Scale.

^#^ One‐way ANOVA test.

There is a significant negative correlation between anxiety and both left‐ and right‐hand NCS, whereas the correlation was insignificant for the depression scale (Table 5).

**TABLE 5:** Correlations between psychological factors and EDX findings

Variables	*r* (Spearman correlation)
HADS‐anxiety vs. left EDX	−0.277
HADS‐anxiety vs. right EDX	−0.240
HADS‐depression vs. left EDX	−0.152
HADS‐depression vs. right EDX	−0.084

Abbreviations: EDX, electrodiagnostic; HADS, Hospital Anxiety and Depression Scale.

Correlations between BCTQ, HADS, DAR‐5, NCV, and DML are shown in Table 6.

**TABLE 6:** Correlations between psychological factors, BCTQ questionnaire, and nerve conduction study

Variables	*r* (Pearson correlation)	*p*‐Value
BCTQ SSS vs. HADS‐anxiety	0.295	**.001**
BCTQ SSS vs. HADS‐depression	0.287	**.001**
BCTQ SSS vs. DAR‐5 anger	0.284	**.002**
BCTQ FSS vs. HADS‐anxiety	0.297	**.001**
BCTQ FSS vs. HADS‐depression	0.293	**.001**
BCTQ FSS vs. DAR‐5 anger	0.181	**.047**
HADS‐anxiety vs. left sensory NCV	0.246	**.008**
HADS‐anxiety vs. left DML	−0.186	**.044**
HADS‐depression vs. left sensory NCV	0.192	**.038**
HADS‐depression vs. left DML	−0.171	.066
DAR‐5 anger vs. left sensory NCV	0.150	.107
DAR‐5 anger vs. left DML	−0.083	.373
HADS‐anxiety vs. right sensory NCV	0.183	.052
HADS‐anxiety vs. right DML	−0.206	**.029**
HADS‐depression vs. right sensory NCV	0.071	.455
HADS‐depression vs. right DML	−0.102	.284
DAR‐5 anger vs. right sensory NCV	0.121	.201
DAR‐5 anger vs. right DML	−0.107	.258

Nerve conductive study was conducted in only case group.

Significant values were in bold font.

*Abbreviations*: BCTQ FSS, Boston Carpal Tunnel Questionnaire Function Status Scale; BCTQ SSS, Boston Carpal Tunnel Questionnaire symptom Severity Scale; DAR‐5, dimensions of anger reaction 5; DML, distal motor latency; HADS, Hospital Anxiety and Depression Scale; NCV, nerve conduction velocity.

When using forward linear regression to regress anxiety score on case–control groups, education and working status, case control was significant with *p* < .001 and *R* ^2^ of 6.2% and work with *p* = .004 and *R* ^2^ of 9.4%. When using the same methods to regress depression score, case–control was significant with *p* < .001 and *R* ^2^ of 7.8%, work with *p* = .004 and *R* ^2^ of 10.9%, and education with *p* = .022 and *R* ^2^ of 12.9%. However, when using the same method to regress anger, only work was significant with *p* = .025 and *R* ^2^ of 1.7%.

## 4 DISCUSSION

This study found a strong association between CTS symptoms severity along with functional status and anxiety, depression, and anger. In regard to anger, this study surprisingly revealed that both cases and controls have problematic anger, and no significant difference between them was found. Furthermore, there was no correlation between anger and NCS. However, regarding anxiety and depression, controls have more normal and milder anxiety and depression when compared to cases of CTS who had more severe anxiety and depression. Anxiety was correlated with milder NCS as it had an inverse correlation with DML in both hands and a positive correlation with sensory NCV in the left hand only. On the other hand, depression had a weak correlation with NCS as it does not relate to DML, and its positive correlation with sensory NCV was only significant to left hands.

This study confirms previous findings (Beleckas et al., 2018; Filho et al., 2020Moghadam‐Ahmadi et al., 2017) that anxiety and depression were more prevalent in CTS patients, but this finding should be interpreted with caution as these studies used different tools other than HADS to either calculate or estimate the percentage of prevalence. For instance, Filho et al. (2020) used DSM‐5 criteria to confirm the diagnosis of anxiety and depression, Moghadam‐Ahmadi et al. (2017) administered Beck Anxiety Inventory (BAI), and Beleckas et al. (2018) used Patient‐Reported Outcomes Measurement Information System (PROMIS). In addition, the prevalence of anxiety and depression in CTS patients was higher than that reported by Filho et al. (2020) and Beleckas et al. (2018); this may be explained by the war environment of Syria, where the study took place.

Our study is consistent with previous research (Sun et al., 2019) regarding the strong association between CTS symptoms severity along with functional status and anxiety and depression. However, these results differ slightly from an earlier study reported by Shin et al. (2018) who states that only CTS symptoms severity is associated with depression and anxiety. Shin et al. (2018)also interestingly reports that depression lessened following the improvement of CTS symptoms. Our study disagrees with Moghadam‐Ahmadi et al.'s (2017) and Papadopoulou et al.'s (2021) study findings disproving the association, too.

Although it is believed that having more severe symptoms is associated with a greater underlying pathophysiology, this is mostly not true with pain (Nunez et al., 2010) since many studies demonstrate that psychological distress act synergistically on pathophysiology at a personal level, and there is a positive correlation between this distress and CTS, as well (Mansfield et al., 2018). Since association does not mean causation, the limitation of our study is that it does not investigate whether CTS causes psychological distress or vice versa is true.

There was an inverse association between anxiety and EDX findings for both right and left hands in terms of general grading. This correlation demonstrates the association between the high grade of anxiety and the low grade of EDX.

The inverse association between anxiety and DML indicates that anxiety is more associated with normal to mild electrodiagnostic CTS grades than moderate to severe grades. The positive correlation between anxiety and sensory NCV in the left hand also supports the mentioned association. This is consistent with other case‐control study's results that found patients with only CTS symptoms without electrodiagnostic (EDX) conformation had the highest mean of HADS anxiety score. However, this study reported that patients with normal EDX have also the highest HADS depression score in contrast to our study, in which depression was not significantly correlated with NCS except for the left hand conduction velocity (McCallum et al., 2019). Psychological factors were related to clinical scales but not EDX results in other research studies (Khan et al., 2017; Papadopoulou et al., 2021). The specific correlation between anxiety and the left hand may shed the light on the psychometric origin of these symptoms. While CTS is known to be most prominent in the dominant hand, this phenomenon may be explained by the idea that symptoms in the left hand may be due to the anxiety itself rather than median nerve injury.

Psychological support should be offered for CTS patients with mild NCS in case positive anxiety and depression were found (Khan et al., 2017).

Research on anger's relationship with CTS lags far behind studying that of anxiety and depression with CTS. To the best of our knowledge, there are no studies in the literature that explore the association of self‐reported anger with symptom severity and functional status in patients with CTS. Our study reveals that both case and control groups have problematic anger, which may be attributable to the traumatic war environment in Syria (Kakaje et al., 2021). There were significant positive correlations between anger and both symptom severity and functional status in our study. In general, the association between pain and anger was highly reported in previous studies. Anger exacerbates pain intensity in both, chronic and acute conditions (Estlander et al., 2008; Greenwood et al., 2003), and vice versa was true as a systematic review stated that patients experiencing chronic pain reported higher degrees of anger in comparison to controls (Sommer et al., 2019). This study provides new insight into anger as its association with CTS is never studied before.

Our study did not come with any limitations. The questionnaires of assessing psychological status cannot substitute clinical diagnosis by medical professionals. Having other factors such as work and education could also affect the results. Finally, a larger sample size could also help determine the differences between the groups, which was not feasible in our study.

In conclusion, this is the first study in Syria about CTS and its association with mental health. There was a strong association between psychological aspects of anger, anxiety, and depression and symptoms severity and functional status of CTS patients. Having CTS symptoms with normal NCS was associated with higher anxiety and depression score. These findings may suggest that being in the war with major distress such as COVID‐19 might indirectly increase CTS symptoms. These findings emphasize the importance of psychological aspects when having hand pain or CTS symptoms. Further studies are needed to evaluate the association between anger and CTS symptoms.

## FUNDING INFORMATION

We received no funding in any form.

## CONFLICT OF INTEREST

The authors declare no conflict of interest.

## AUTHOR CONTRIBUTION

**Aya Alsharif**: Conceptualization; data curation; formal analysis; investigation; methodology; project administration; resources; software; validation; original draft; writing – review and editing. **Aya Al Habbal**: Conceptualization; data curation; formal analysis; investigation; methodology; project administration; resources; software; validation; original draft; writing – review and editing. **Yaman Daaboul**: Software; resources; conceptualization; project administration; supervision; validation. **Lama Al Hawat**: Software; investigation; project administration. **Osama Al Habbal**: Software; investigation; project administration. **Ameer Kakaje**: Conceptualization; formal analysis; methodology; project administration; supervision; resources; validation; original draft; writing – review and editing.

### PEER REVIEW

The peer review history for this article is available at https://publons.com/publon/10.1002/brb3.2493

## Data Availability

Data will be made available upon reasonable request
